# A Review of Anti-Angiogenic Targets for Monoclonal Antibody Cancer Therapy

**DOI:** 10.3390/ijms18081786

**Published:** 2017-08-17

**Authors:** Deok-Hoon Kong, Mi Ra Kim, Ji Hye Jang, Hee-Jun Na, Sukmook Lee

**Affiliations:** Research Center, Scripps Korea Antibody Institute, Chuncheon 200-701, Korea; kong0131@skai.or.kr (D.-H.K.); cslove526@skai.or.kr (M.R.K.); jjh717@skai.or.kr (J.H.J.)

**Keywords:** antibody, cancer therapy, therapeutic target, tumor, tumor angiogenesis, VEGF

## Abstract

Tumor angiogenesis is a key event that governs tumor progression and metastasis. It is controlled by the complicated and coordinated actions of pro-angiogenic factors and their receptors that become upregulated during tumorigenesis. Over the past several decades, vascular endothelial growth factor (VEGF) signaling has been identified as a central axis in tumor angiogenesis. The remarkable advent of recombinant antibody technology has led to the development of bevacizumab, a humanized antibody that targets VEGF and is a leading clinical therapy to suppress tumor angiogenesis. However, despite the clinical efficacy of bevacizumab, its significant side effects and drug resistance have raised concerns necessitating the identification of novel drug targets and development of novel therapeutics to combat tumor angiogenesis. This review will highlight the role and relevance of VEGF and other potential therapeutic targets and their receptors in angiogenesis. Simultaneously, we will also cover the current status of monoclonal antibodies being developed to target these candidates for cancer therapy.

## 1. Introduction

Angiogenesis is a physiological process in which new blood vessels are formed from pre-existing vessels. It occurs during normal growth and development, as well as during wound healing [1,2,3]. Under physiological conditions, angiogenesis is tightly regulated by the complex and coordinated actions of pro-angiogenic and anti-angiogenic factors according to the spatiotemporal requirements of cells or tissues [4]. To date, many pro-angiogenic factors and their cognate receptors have been identified, including vascular endothelial growth factor (VEGF), platelet-derived growth factor (PDGF), angiopoietin (Ang), hepatocyte growth factor (HGF), and epidermal growth factor (EGF) [5,6,7,8,9,10].

Tumor angiogenesis is a hallmark of cancer and plays a crucial role in providing oxygen and nutrients to tumor cells during cancer progression and metastasis [11,12]. Under pathological conditions, many pro-angiogenic factors and their receptors are upregulated; among these factors, VEGF is generally regarded as a key regulator of tumor angiogenesis [13,14,15]. Bevacizumab, an anti-VEGF antibody, was recently developed as a cancer therapy to suppress tumor angiogenesis [16,17]. Although bevacizumab is clinically effective for treating patients with a variety of cancers, it has frequent complications due to its inhibition of VEGF signaling in normal endothelial cells, which express high levels of VEGF receptors (VEGFRs). Importantly, bevacizumab treatment is associated with severe side effects, including bleeding, proteinuria, hypertension, gastrointestinal perforation, and stroke [18,19]. Furthermore, in glioblastoma multiforme patients, bevacizumab treatment is associated with the presence of tumor cells that have an infiltrative phenotype, and high levels of matrix metalloproteinase (MMP)-2 and membrane-type 1 MMP [20,21,22]. In addition, long-term bevacizumab treatment can lead to the development of drug resistance, due to the upregulation of other redundant tumor-derived angiogenic factors, including Ang, EGF, HGF, and PDGF [23,24,25].

**Figure 1:**
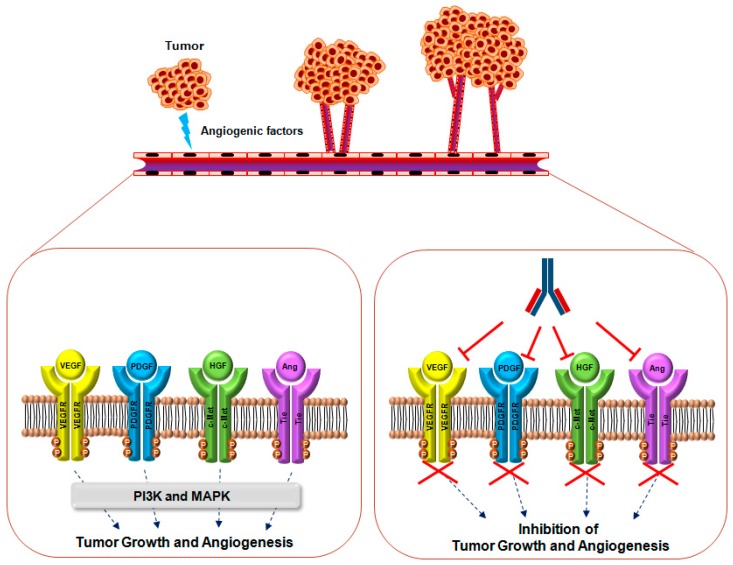
Mechanisms of action of monoclonal antibodies targeting VEGF, PDGF, HGF, Ang, and their receptors for suppressing tumor growth and angiogenesis. Under pathological conditions, including hypoxia, most tumor cells and/or adjacent cells upregulate the expression of many angiogenic factors, including VEGF, PDGF, HGF, and Ang, and secrete them within the tumor microenvironment. When these molecules bind their cognate receptors, receptor dimerization and autophosphorylation stimulate downstream signaling molecules including phosphatidylinositol-4,5-bisphosphate 3-kinase (PI3K)/v-Akt murine thymoma viral oncogene (Akt) and MAPK (dash lines) for the promotion of tumor growth and angiogenesis. Currently, most antibody therapeutics are being developed to block the interaction between agonists and their receptors (T arrows).

**Figure 2:**
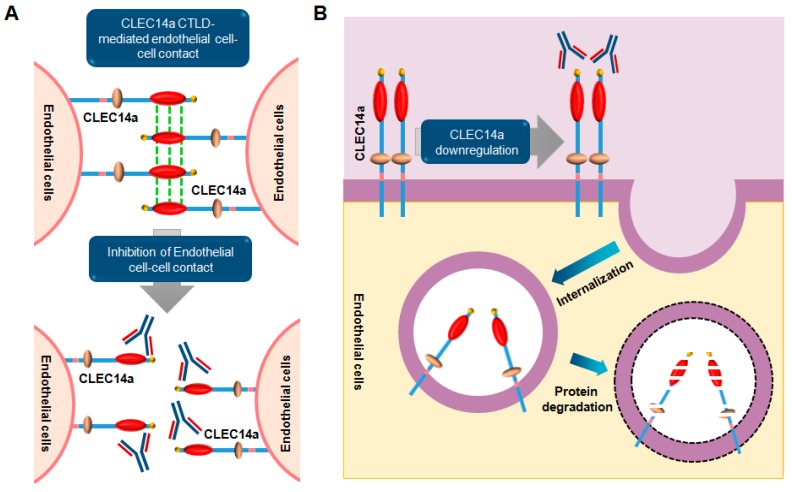
Dual mode of action of a monoclonal antibody targeting the CLEC14a (C-type domain family 14 member) CTLD (C-type lectin-like domain) in cancer. (**A**) In tumor angiogenesis, the CLEC14a CTLD (red) plays a key role in mediating endothelial cell-cell contacts. When a targeting antibody binds to the CTLD of CLEC14a on tumor vessels, endothelial cell-cell contacts are inhibited, resulting in inhibition of tumor angiogenesis; (**B**) Simultaneously, binding of the antibody to CLEC14a on endothelial cells induces internalization of CLEC14a and eventually leads to its downregulation. These molecular mechanisms are important for suppressing CLEC14a-mediated tumor angiogenesis.

Despite the wide-range of clinical usefulness of bevacizumab for cancer therapy, the identification of novel angiogenic therapeutic targets and development of novel drugs as alternative or combination treatments with existing drugs are needed to improve the survival and quality of life of cancer patients. In the present article, we review the role and relevance of VEGF/VEGFR and four other therapeutic targets in tumorigenesis, as well as the current status (Table 1) and mechanisms of action of therapeutic antibodies being developed for anti-angiogenic therapy (Figure 1 and Figure 2).

## 2. VEGF Signaling

### 2.1. Overview

VEGF, first identified as a factor that promotes vascular permeability and vascular endothelial cell mitosis in the 1980s, is a key player in angiogenesis, as well as in endothelial proliferation, migration, and nitric oxide (NO) release [26,27,28]. The mammalian VEGF proteins are dimeric glycoproteins with a molecular weight of approximately 40 kDa. Although VEGF-A is the most well characterized VEGF isoform, the VEGF family consists of five distinct isoforms: VEGF-A, VEGF-B, VEGF-C, VEGF-D, and placenta growth factor (PlGF). Structurally, VEGF proteins have eight regularly spaced cysteine residues, which form intramolecular disulfide bonds that generate three loops; two intermolecular disulfide bonds allow for a homodimer structure between two VEGF molecules [29,30]. In addition, VEGF has various alternative splice variants, which exhibit different binding affinity for VEGFR co-receptors, including neuropilins and heparin sulfate proteoglycans [31]. For instance, VEGF-A, VEGF-B, and PlGF can be divided into five (VEGF-A121, VEGF-A145, VEGF-A165, VEGF-A189, and VEGF-A206), two (VEGF-B167 and VEGF-B186), and four (PlGF-1, PlGF-2, PlGF-3, and PlGF-4) variants [32,33,34]. Among the VEGF-A variants, VEGF-A165 and VEGF-A189 bind neuropilins and heparin sulfate proteoglycans, whereas VEGF-A121 does not bind either [31]. Importantly, this molecular diversity alters bioavailability and activity, which in turn, allows for the initiation of various cellular responses.

VEGF-mediated cellular functions occur via the activation of three receptors: VEGFR-1 (Flt-1), VEGFR-2 (KDR/Flk-1), and VEGFR-3 (Flt-4). VEGFRs are members of the receptor tyrosine kinase (RTK) superfamily and are composed of an extracellular domain with seven immunoglobulin (Ig)-like domains, a transmembrane domain, a juxtamembrane domain, and an intracellular domain with a split tyrosine kinase domain and C-terminal tail [35]. Ig-like domains are involved in ligand binding; in particular, Ig-like domains 2 in VEGFR-1 and Ig-like domains 3 in VEGFR-2 are associated with ligand-binding site and ligand-binding specificity, respectively [32]. Upon ligand binding, VEGFRs form homo- and heterodimers, which induce tyrosine transphosphorylation of the intracellular kinase domains and activates signal transduction.

VEGF signaling is one of the major signaling pathways required for embryonic vascular development and angiogenesis. VEGF-A, VEGF-B, and P1GF are constitutive VEGFR-1 agonists that induce migration and proliferation. In endothelial cell and macrophages, VEGFR-1 initiates migration by stimulating the phosphatidylinositol-4,5-bisphophate 3-kinase (PI3K)/Akt-Rac1 pathway through a receptor for activated protein kinase C 1 [36]. PI3K pathway activation by VEGFR-1 is also linked to endothelial cell proliferation and tubulogenesis [37]. In addition, stimulation of monocyte VEGFR-1 induces migration through the activation of several intracellular signaling molecules, including PI3K, Akt, extracellular signal–regulated kinases (ERK)1/2, and p38 mitogen-activated protein kinase (MAPK) [38]. In particular, VEGF-A activation of VEGFR-2 initiates PLCγ interaction with the Tyr1175 residue of internalized VEGFR-2 in early endosome antigen 1-positive endosomes [32,39]. This leads to subsequent cascades that activate Ca^2+^-dependent rapidly accelerated fibrosarcoma (RAF)-mitogen-activated protein kinase kinase (MEK)-ERK1/2 and calmodulin-calcineurin-nuclear factor of activated T cells pathways, which together promote endothelial cell migration, proliferation, and homeostasis [32,39]. The Tyr951 residue of VEGFR-2 also provides a binding site for the v-sarcoma viral oncogene homolog (SRC) homology 2 (SH2) domain of T-cell-specific adaptor protein [32,39,40]. Interaction of T-cell-specific adaptor protein with VEGFR-2 leads to the activation of Akt signaling, which determines endothelial cell shape, adhesion, survival, and vessel permeability [32,39,40]. In addition, Akt activation by VEGFR-2 stimulates endothelial nitric oxide synthase, inducing release of NO into the extracellular space, which promotes vasodilation in adjacent smooth muscle cells [39]. VEGF-C or VEGF-D activation of VEGFR-3 plays a critical role in lymphangiogenesis; in lymphatic endothelial cells, SHC and growth factor receptor bound protein 2 (GRB2) adaptor proteins are recruited to activated VEGFR-3 and promote ERK1/2 and PI3K/Akt pathway, which is critical for lymphatic endothelial cell migration [41,42].

### 2.2. Relevance of VEGF and VEGFR in Cancer

VEGF/VEGFR signaling is critical for vessel development during embryogenesis and tumorigenesis [43,44]. In particular, VEGF-A and its receptors have been well studied in physiological and pathological neovascularization processes, such as tumor angiogenesis. Several studies have demonstrated that VEGF-A is induced by exposure to hypoxic conditions and secreted by tumor cells and tumor-associated stroma [45,46,47]. VEGFRs are also present in both liquid and solid tumors, such as leukemia, non-small cell lung cancer (NSCLC), gastric cancer, and breast cancer [48,49,50,51]. In NSCLC, the five-year survival rates of patients expressing low and high *VEGF* mRNA were 77.9% and 16.7%, respectively [52]. Xenograft mouse models of VEGF-A-deficient lung carcinoma display delayed tumor growth and reduced tumor weight, as well as the inhibition of angiogenesis and induction of apoptosis [49]. In breast cancer, VEGF-A has been reported to promote the proliferation, survival, and metastasis of breast cancer cells in vitro and in vivo. VEGF-A expression in breast cancer cells, including MCF-7, BT-474, and T47-D cells, induces proliferation and survival in vitro through Bcl-2, an anti-apoptotic protein [50]. Additionally, inhibition of VEGF-A in a xenograft mouse model of murine breast carcinoma 4T1 cells suppresses primary tumor growth and prevents pulmonary metastases [53].

In addition to NSCLC and breast cancer, overexpression of VEGF-A and VEGF-C occurs in gastric cancer. In gastric cancer patients, VEGF-A and VEGF-C are associated with large tumor size, higher peritumoral lymphatic vessel density, microvessel density, lymphatic vessel invasion, lymph node metastasis, and worse prognosis [51]. Furthermore, silencing of VEGF-A and VEGF-C significantly inhibits cell proliferation, tumor growth, and tumor size in vitro and in vivo [51]. The tumor-promoting effects of VEGF/VEGFR have been demonstrated in other cancer types, including neuroblastoma, prostate cancer, and hepatocellular carcinoma [54,55,56]. Together, these studies suggest that the VEGF signaling axis is involved in multiple aspects of cancer development and may be a good prognostic and therapeutic target for cancer patients.

### 2.3. Monoclonal Antibodies Targeting VEGF and VEGFR

Monoclonal antibody-based therapy is one of the most important strategies for treating patients with various diseases, including solid tumors, hematological malignancies, immunological disorders, and eye diseases [57,58,59]. So far, there are more than 40 therapeutic antibodies approved by the United States Food and Drug Administration (US FDA) for various indications, and many more are currently being evaluated in clinical trials [60].

Bevacizumab (Avastin^®^, Genentech, San Francisco, CA, USA) is a recombinant humanized immunoglobulin G (IgG) monoclonal antibody that targets VEGF-A and inhibits formation of the VEGF-A and VEGFR-2 complex [61]. In 2004, bevacizumab was first approved by the US FDA to treat metastatic colorectal cancer in combination with standard chemotherapy [62]. Currently, it is widely used to treat various cancers, including metastatic non-squamous NSCLC, metastatic renal cell carcinoma, breast cancer, epithelial ovarian cancer, and glioblastoma [63,64,65,66,67]. Due to the clinical validation of bevacizumab as a specific inhibitor of interaction between VEGF-A and VEGFR2, the increasing numbers of monoclonal antibodies in development has targeted VEGR2 as a promising molecular target for anti-angiogenesis.

Aflibercept (Zaltrap^®^, Regeneron pharmaceuticals, Tarrytown, NY, USA) is an Fc fusion protein consisting of the extracellular domains of VEGFR-1 and VEGFR-2 fused with the Fc domain of human IgG [68]. It acts as a VEGF trap that inhibits the activity of VEGF isoforms, including VEGF-A, VEGF-B, and PlGF, and suppresses tumor angiogenesis. In 2012, the US FDA approved aflibercept to treat patients with metastatic colorectal cancer that is resistant to or has progressed following an oxaliplatin-based regimen [69]. Aflibercept (Eylea) was also approved by the US FDA in 2011 for the treatment of wet age-related macular degeneration, the leading cause of blindness in the elderly [70,71]. Furthermore, although bevacizumab is not approved by the FDA for this indication, it has been prescribed off-label because of its cost effectiveness and significant inhibitory effect on VEGF-dependent neovascularization [72,73].

Ramucirumab (Cyramza^®^, ImClone Systems Incorporated, Bridgewater, NJ, USA) is a fully human monoclonal antibody that specifically targets VEGFR2 by blocking its interaction with VEGF ligands [74,75,76]. In 2014, the US FDA approved ramucirumab for the treatment of advanced gastric or gastro-esophageal junction adenocarcinoma and metastatic non-small-cell lung carcinoma [77,78]. Unfortunately, ramucirumab has not been significantly effective in clinical trials for breast and liver cancer, although it is currently in a phase III trial for locally advanced or metastatic urothelial carcinoma [79,80,81].

Tanibirumab is a fully human monoclonal antibody developed by PharmAbcine (Daejon, Korea). It specifically binds VEGFR-2 and blocks binding of VEGFR ligands, including VEGF-A, VEGF-C, and VEGF-D [82]. In 2013, a phase I trial of tanibirumab in patients with solid tumors refractory to standard chemotherapy was finished with good safety and efficacy results [83].

## 3. PDGF Signaling

### 3.1. Overview

In the 1970s, PDGF was first isolated from platelet extracts and identified as a mitogenic factor in in vitro cultures of mammalian cells, including fibroblasts, smooth muscle cells, and glial cells [84,85]. Heldin et al. reported that purified PDGF stimulates tyrosine kinase activity in membranes prepared from human fibroblasts, suggesting that PDGFR had tyrosine kinase activity [86].

PDGFs are assembled in the endoplasmic reticulum as inactive precursor disulfide-linked homodimers composed of AA-, BB-, CC-, and DD-polypeptide chains, and the heterodimer PDGF-AB [87]. Generally, the structure of PDGF family members consists of a growth factor domain of about 100 amino acid residues that is involved in receptor-binding and dimerization and a prodomains of various length that is an attached amino acid sequence found in N-terminal extension [88,89,90]. These isoforms are activated by proteolytic processing: PDGF-AA, -AB, and -BB have short N-terminal extensions that are cleaved by intracellular proteolysis in secretory vesicles, whereas PDGF-CC and -DD have a distinct protein domain called the CUB-domain, which is cleaved by extracellular proteolysis [87]. Active isoforms of PDGF bind to the α- and β-RTKs, PDGFRα and PDGFRβ, and induce the activation of downstream signaling pathways following tyrosine autophosphorylation [84].

PDGFRs are transmembrane proteins with intrinsic tyrosine kinase activity. The two PDGFRs are composed of an extracellular domain with five Ig-like domains, a transmembrane domain, and an intracellular portion that contains the kinase domain [89]. PDGFRs are activated by ligand binding to Ig-like domains 2 and 3, which are further stabilized by dimerization, resulting in direct receptor-receptor interactions involving Ig-like domain 4 [91,92]. Upon ligand binding, PDGFR dimerization induces the transphosphorylation of the cytoplasmic catalytic domain, where tyrosine residues serve as docking sites for downstream molecules, which include members of the MAPK and PI3K signaling pathways [93].

PDGFR activation of the rat sarcoma (RAS)-MAPK pathway is initiated when the SH2 domain of GRB2 binds to a PDGFR phosphotyrosine residue, forming a complex with Son of sevenless homology 1 (SOS1) through its SH3 domains [93]. Son1 in turn induces the downstream activation of RAF-1 and MAPK cascades by activating RAS, leading to cell growth, differentiation, and migration [93]. PDGF stimulates the PI3K pathway through the activation of serine/threonine kinases, such as Akt/PKB, PKC, P70 S6 kinase, JNK, and small GTPases of the Rho family, leading to actin reorganization, directed cell movements, stimulation of cell growth, and inhibition of apoptosis [93,94,95,96,97,98].

### 3.2. The Role and Relevance of PDGF and PDGFR in Cancer

Signal transduction via PDGF and PDGFR has been implicated in several physiological and pathological processes, including wound healing, embryogenesis, bone formation, and tumor growth [93,99,100,101,102]. PDGF and PDGFR signaling is also associated with the normal development of the kidney, brain, and respiratory systems [103]. Under physiological conditions, endothelial-derived PDGF-BB activates pericytes and smooth muscle cells through paracrine signals, which in turn, affects vascular remodeling, maturation, and stability. PDGF-BB also modulates endothelial proliferation, migration, and tube formation, and contributes to angiogenesis in vitro; in contrast, PDGF-AA has no such effect or induces a weak angiogenic response [84]. PDGF-CC promotes the angiogenesis of the mouse cornea through PDGFRαα and -αβ dimers [104]. In addition, PDGF-CC is abundantly expressed in angiogenic tissues, such as the placenta, ovary, and embryo [105,106]. Finally, PDGF-DD plays an important role in increasing interstitial fluid pressure, macrophage recruitment, and blood vessel maturation during angiogenesis in the skin and skeletal muscles [107]. Interestingly, inhibition of PDGF-DD also suppresses ocular neovascularization [108].

As mentioned above, PDGF and PDGFR play important roles in vascular remodeling, maturation, and stability during angiogenesis. Since angiogenesis is crucial for tumor growth and metastasis, the relationship between PDGF and PDGFR and carcinogenesis has been studied for several decades. Overexpression of PDGFR is mainly observed in colorectal cancers and is closely associated with angiogenesis, invasion, and metastasis [109]. Its overexpression also correlates with poor patient prognosis [109]. In addition to colorectal cancers, overexpression of PDGF and PDGFR occurs in brain tumors like glioblastoma. In particular, PDGF-CC affects the maturation and stabilization of blood vessels in a xenograft mouse model of VEGF-CC-overexpressed human glioblastoma and these functions are weakened in response to anti-VEGF therapy [110]. Furthermore, PDGF-DD and PDGFRβ are upregulated in most primary and metastatic prostate cancer cells. Overexpression of PDGF-DD in a xenograft mouse model of prostate cancer and in PC3 prostate cancer cells promotes invasiveness and epithelial-mesenchymal transition, respectively [87]. Moreover, siRNA-mediated knockdown of PDGFRα and PDGFRβ suppresses prostate cancer cell growth by the suppression of angiogenesis in a prostate cancer xenograft model [87,111]. The PDGF/PDGFR pathway also plays a role in transforming growth factor-β-mediated hepatocellular carcinoma and epithelial-mesenchymal transition-mediated breast cancer progression [112,113].

Small molecule compounds targeting PDGFRs have been intensively investigated and include imatinib, sunitinib, regorafenib, and pazopanib [87]. These inhibitors suppress the activation of PDGFRs, as well as other kinases, such as Abl kinase, Kit, VEGFR, Raf, and FGFR [87]. Currently, they are approved in the clinic for first- or second-line treatment of various cancers, including metastatic colorectal cancer, metastatic renal cell carcinoma, and gastrointestinal stromal tumors [114]. However, the broad range of toxicity and side effects due to off-target effects of these compounds are major obstacles yet to be overcome.

### 3.3. Monoclonal Antibodies Targeting PDGF and PDGFR

PDGF and PDGFR are overexpressed in many human tumor types and contribute to cancer progression by promoting blood vessel maturation and angiogenesis, resulting in poor response to chemotherapy and decreased patient survival. Olaratumab (LARTRUVO^®^, Eli Lilly, Indianapolis, IN, USA) is a fully human monoclonal antibody that targets human PDGFRα with high affinity (Kd = 40 pM) [115,116]. It blocks ligand binding to PDGFRα, inhibits tyrosine phosphorylation of the receptor, and suppresses ligand-induced phosphorylation of downstream signaling molecules [116]. In 2005, Loizos et al. first developed olaratumab using transgenic mice to produce fully human antibodies [115]. In preclinical studies, olaratumab reduced tumor growth of human glioblastoma and leiomyosarcoma [115]. In a prostate cancer xenograft model, targeting PDGFRα with olaratumab delayed the progression of early skeletal metastatic foci and reduced the size of skeletal tumors [117]. Olaratumab had also no apparent adverse effects when administered to cynomolgus monkeys at doses up to 75 mg/kg per week [118]. Based on its safety and anti-tumor activity in preclinical studies, olaratumab entered clinical development. In a phase I study in patients with advanced solid tumors, olaratumab had no dose-limiting toxicities and significant anti-tumor activity [118]. Given these results and the improvement of median survival and progression-free survival, the US FDA granted accelerated approval to olaratumab in 2016 for the treatment of patients with soft tissue sarcoma that is not amenable to curative treatment with radiotherapy or surgery [119].

## 4. Ang Signaling

### 4.1. Overview

Ang and its receptor, tyrosine kinase with Ig-like and EGF-like domains (Tie) receptor, comprise a major signaling node that modulates vascular development and remodeling [120]. Angs are ~70 kDa glycoproteins consisting of four distinct isoforms: Ang-1, Ang-2, Ang-3, and Ang-4. Ang-1 and Ang-2 were first isolated using a secretion-trap expression cloning and low stringency DNA homology cloning approach [121,122]. Subsequently, Ang-3 and Ang-4 were identified as the mouse and human orthologues, respectively [121]. Among these members, Ang-1 and Ang-2 share approximately 60% amino acid sequence identity, and are expressed in the vascular endothelium [123]. Ang-3, the mouse orthologue of Ang-4, is expressed in a number of murine tissues, and Ang-4 is highly expressed in human lung [121,124].

In the early 1990s, Tie receptors were first identified as endothelial cell RTKs. There are two Tie receptors, Tie-1 and Tie-2. Ang directly binds Tie-2, while Tie-1 lacks a ligand [121,122,125,126]. Tie-1 and Tie-2 are composed of an extracellular domain, a transmembrane domain, and an intracellular domain that transduces downstream signaling [127]. At the N-terminus, the extracellular domain has two Ig-like domains for Ang binding, followed by EGF-like domains, another Ig-like domain, and three fibronectin type III-like domains [127]. The intracellular domain possesses a split tyrosine kinase domain and carboxy-terminal tail.

The binding of Ang to Tie-2 leads to receptor dimerization and autophosphorylation at five tyrosine residues in the intracellular kinase domain, resulting in vessel remodeling and vessel maturation during embryonic development and adult vessel homeostasis [8,128]. Ang-1 is a constitutive agonist of Tie-2 for endothelial cell survival, whereas Ang-2 is generally considered to be an antagonist that competes with Ang-1 for Tie-2 binding; both Ang-1 and Ang-2 have comparable binding affinities for Tie-2 [123,129,130]. Upon binding of Ang-1, Tie-2 induces a number of intracellular signaling pathways, including PI3K and MAPK. The p85 subunit of PI3K interacts with Tyr1101 of activated Tie-2 and stimulates the PI3K-Akt pathway, which promotes endothelial cell survival and NO release [130,131]. This occurs through the inhibition of Smac, a protein that stimulates cytochrome c-dependent caspase activation and release from mitochondria [132]. This, in turn, increases the expression of survivin and activation of endothelial nitric oxide synthase [132]. This pathway also inhibits forkhead transcription factor FKHR and prevents endothelial cell death [133].

The RAS-RAF-MAPK pathway is also initiated in endothelial cells by the interaction of activated Tie-2 with the SH2 domain of GRB2, which initiates the synthesis of platelet activating factor, anti-inflammatory responses, migration, proliferation, permeability, and morphogenesis [130,132]. Cell migration is stimulated when the downstream of kinase-related docking protein is recruited to Tie-2 and interacts with the non-catalytic region of the tyrosine kinase Nck and serine/threonine p21-activated kinase [130,132,134]. Tie-2 also mediates anti-inflammatory and anti-apoptotic activity by interacting with the A20-binding inhibitor of nuclear factor-kB-2 [135,136].

### 4.2. The Role and Relevance of Ang and the Tie Receptor in Cancer

Ang/Tie signaling is a fundamental process for proper embryonic development. The overexpression of Ang-2 and a potent Ang-1 variant (COMP-Ang-1) in a hindlimb ischemic model and hypercholesterolemic mouse model induces postnatal and cavernous angiogenesis, demonstrating that Ang/Tie signaling is an essential regulator of vascular development under physiological conditions [137,138].

Under pathological conditions, such as cancer, Ang/Tie signaling plays important and rate-limiting roles in the early stages of tumor vascularization. The roles of Ang-3 and Ang-4 in pathological conditions are relatively unknown, whereas many studies have demonstrated the importance of Ang-1 and Ang-2 in tumor growth and tumor-associated angiogenesis. Ang-2 is widely expressed in various cancers including stomach, colon, hepatocellular carcinoma, melanoma, and NSCLC, and plays a distinct role in tumor pathogenesis [139,140,141,142,143]. In an Ang-2-transfected colon cancer xenograft model, Ang-2 led to increased angiogenesis and tumor growth [141]. Moreover, the forced expression of Ang-2 promotes metastasis, while Ang-2 inhibition represses tumor angiogenesis and metastasis in mouse models of spontaneous carcinogenesis [144,145]. In addition, Ang-2-deficiency in mice implanted with lung carcinoma and melanoma retards tumor growth and increases tumor vascular maturation and pericyte coverage compared to wild-type mice [146]. In contrast to the tumor-promoting effects of Ang-2, the role of Ang-1 in cancer is controversial. Forced expression of Ang-1 increases tumor growth in a model of rat glioma, as well as in animal models of plasma cell tumor [147,148]. In contrast, Ang-1 overexpression reduces tumor growth in multiple other cancers, including squamous cell carcinoma, breast cancer, and colon cancer [149,150,151]. These results suggest that modulation of the Ang-2 to Ang-1 ratio may be important for the treatment of tumor growth and angiogenesis.

### 4.3. Monoclonal Antibodies Targeting Ang and Tie Receptors

Ang/Tie signaling is a key regulator of tumor vascular remodeling. Generally, it is thought that Ang-1 mediates vessel stabilization and maturation, whereas Ang-2 induces vessel destabilization and permeability. Ang-2 is upregulated in a number of human cancers, and high levels of circulating Ang-2 are associated with a poor prognosis in breast cancer, gastric cancer, hepatocellular carcinoma, and lung cancer [152].

Nesvacumab is a fully human monoclonal antibody developed by Regeneron Pharmaceuticals (Tarrytown, NY, USA). It selectively binds Ang-2 with high affinity (Kd = 24 pM) and blocks Ang-2 binding to Tie-2 [153]. In preclinical studies, nesvacumab significantly inhibited tumor growth in xenograft models of prostate adenocarcinoma, colorectal adenocarcinoma, and epidermoid carcinoma [153,154]. Nesvacumab also had no apparent adverse toxic effects in Sprague Dawley rats and cynomolgus monkeys [153]. On the basis of its safety and anti-tumor activity in preclinical studies, nesvacumab entered phase I trials for advanced solid tumors, which reported that nesvacumab had anti-tumor activity within an acceptable safety profile [153]. In addition, the combination trials of nesvacumab with aflibercept have completed enrollment (Clinical trials information: NCT01688960).

AMG780 is a fully human monoclonal antibody developed by Amgen (Thousand Oaks, CA, USA) that binds to Ang-1 and Ang-2 [155]. It was isolated from a human antigen-binding fragments (Fab) library using bio-panning with phage display technology. Currently, phase I trials in patients with advanced solid tumors have been completed; the results demonstrate that AMG 780 can be administered at doses up to 30 mg/kg every two weeks, with anti-tumor effects in six of 31 evaluable patients [156].

Vanucizumab is a humanized bispecific antibody developed by Genentech (San Francisco, CA, USA) that specifically binds to Ang-2 and VEGF-A [157]. Vanucizumab effectively reduced tumor growth and had anti-angiogenic activity in a xenograft model of colon cancer, and a phase II trial for patients with metastatic colorectal cancers was completed in 2017 (Clinical trials information: NCT02141295) [158]. 

MEDI3617 is a fully human monoclonal antibody developed by Medlmmune LLC (Gaithersburg, MD, USA) that neutralizes Ang-2 by blocking its binding to Tie-2 [159]. In several xenograft models of colorectal carcinoma, renal carcinoma, hepatocellular carcinoma, lung carcinoma, colorectal carcinoma, and ovarian carcinoma, MEDI3617 significantly inhibited tumor growth by the modulation of tumor angiogenesis [159]. Currently, MEDI3617 had an acceptable safety profile in phase I trials for advanced solid tumors (Clinical trials information: NCT01248949).

## 5. HGF Signaling

### 5.1. Overview

In 1984, Nakamura et al. identified a putative hepatotrophic factor, named HGF, in the serum of hepatectomized rats [160]. HGF is secreted by mesenchymal cells as an inert precursor single-chain, and is converted into its bioactive form via cleavage by extracellular proteases, such as urokinase-type plasminogen activator, HGF activator, factors XII and XI, matriptase, and hepsin [161]. It plays a key role in cell proliferation, survival, motility, scattering, differentiation, and morphogenesis [161]. The mature form of HGF, also known as scatter factor, is composed of two subunits (a 69 kDa α-subunit and a 34 kDa β-subunit), which are linked by a disulfide bond [162]. The α-subunit contains an N-terminal hairpin loop followed by four kringle domains, with an 80 amino acid double-looped structure formed by three internal disulfide bridges for each kringle domain [163]. The β-subunit consists of the serine proteinase homology domain, but lacks proteolytic activity [163]. The active form of HGF binds to the c-MET proto-oncogene and induces the phosphorylation of tyrosine residues on c-MET, which initiates signal transduction upon recruitment of downstream adaptor molecules.

c-MET is also a member of the RTK family, and was identified as the HGF receptor in 1992 [164]. It is expressed in epithelial cells of many organs, including the liver, pancreas, prostate, kidney, muscle, and bone marrow [165]. Structurally, c-MET is a heterodimer composed of a 50 kDa α-chain and a 145 kDa β-chain [164]. The extracellular domain of the β-chain has three domains: a semaphorin (Sema) domain, a plexin-semaphorin-integrin domain, and four immunoglobulin-plexin-transcription domains [163]. The N-terminal Sema domain is linked to the α-chain by a disulfide bond that includes the entire α-subunit and part of the β-subunit [163]. The plexin-semaphorin-integrin domain follows the Sema domain and includes four disulfide bonds [163]. The intracellular domain consists of a juxtamembrane domain, a tyrosine kinase catalytic domain, and a C-terminus tail [166]. Phosphorylation of Tyr1234 and Tyr1235 within the kinase domain promotes enzyme activity, whereas phosphorylation of Ser985 and Tyr1003 in the juxtamembrane domain downregulates kinase activity [167]. Other phosphorylation sites in the C-terminal tail, Tyr1349 and Tyr1356, act as multifunctional docking sites for recruitment of signal transducers and adaptors [165].

Like other RTKs, binding of HGF to c-MET leads to receptor homodimerization and autophosphorylation of tyrosine residues within the intracellular domain. This initiates signal transduction through recruitment of adaptor proteins, including GRB2 and SHC, and effector molecules like PI3K and SRC [168,169,170]. MAPK signaling (described above) is one of the main signaling pathways downstream of HGF/c-MET and leads to cell proliferation, motility, and cell cycle progression [163,171]. The PI3K pathway, another HGF/c-MET effector pathway, also mediates cell survival through Akt [172]. In addition, c-MET directly stimulates the SRC-FAK cascades to induce cell migration and promote anchorage-independent growth [170,173,174]. c-MET signaling is also mediated by co-receptors, such as CD44v6, integrin α6β4, and GPCRs that physically associate with c-MET at the cell surface [175,176,177].

### 5.2. The Role and Relevance of HGF and c-MET Signaling in Cancer

HGF is a motility and morphogenic factor. HGF interacts with c-MET via an autocrine and/or paracrine signaling loop and participates in a variety of biological responses, such as embryonic development, epithelial branching morphogenesis, postnatal organ regeneration, wound healing, and tumor development [178]. Zhu et al. first demonstrated that c-MET signaling is essential for HGF-induced motogenic, invasive, and morphogenic responses in Madin-Darby canine kidney epithelial cells [179]. c-MET knockout mice have impeded skeletal muscle formation in the limb and diaphragm; in addition, mice lacking HGF die in utero due to reduced liver size and loss of parenchymal cells [180,181]. Together, these in vivo studies suggest that HGF/c-MET signaling is essential for proper development and multiple biological functions, including cell growth, shape, and movement.

c-MET was first isolated from human osteosarcoma cell lines and originally identified as an oncogene [182]. c-MET signaling is transiently activated in physiological process, but often constitutively activated in tumor cells. Expression of a form of c-MET, containing a translocating promoter region, causes mammary tumor development, as well as the development of other malignancies in transgenic mice [183]. This c-MET translocation has also been found in human gastric cancer precursor lesions [184]. In addition, cell lines that ectopically overexpress c-MET or HGF become transformed and metastasized in nude mice, and similarly, HGF or c-MET transgenic mice develop metastatic tumors [185,186,187]. Abnormal expression of HGF and c-MET has also been found in many kinds of solid cancers, including breast, colon, lung, ovary, kidney, prostate, bladder, and liver, and correlates with metastasis and poor prognosis [178].

### 5.3. Monoclonal Antibodies Targeting HGF and c-MET

HGF/c-MET signaling has been extensively studied as a therapeutic target for various solid tumors over the last few years. Rilotumumab (AMG102) is a fully human monoclonal antibody developed by Amgen (Thousand Oaks, CA, USA) that binds to HGF and blocks binding to c-MET, thereby inhibiting HGF/c-MET-driven signaling [188]. In preclinical studies, rilotumumab showed no toxicities in the cardiovascular, respiratory, or central nervous systems when administered at doses up to 100 mg/kg once weekly for up to one month [189]. It also exhibited multiple anti-tumor effects, including inhibition of tumor growth, induction of tumor regression, initiation of apoptosis, and abrogation of cell proliferation, in xenograft models of human glioblastoma cancer [190]. On the basis of its safety and anti-tumor activity in preclinical studies, rilotumumab entered clinical studies. In phase II clinical trials, patients with gastric and esophagogastric junction cancers treated with rilotumumab plus epirubicin, cisplatin, and capecitabine (ECX) had improved progression-free survival and overall survival compared to the placebo plus ECX group [191]. A phase III clinical trial of rilotumumab in combination with ECX was conducted in patients with gastric and esophagogastric junction cancers and terminated in 2016, due to no statistically significant improvement of overall survival (Clinical trials information: NCT01697072). In addition, many clinical trials of rilotumumab in combination with RTK inhibitors, such as placebo, avastin and erlotinib, have proceeded in patients with NSCLC, metastatic colorectal cancer, prostate cancer, and renal cell carcinoma (Clinical trials information: NCT01233687, NCT00788957, NCT00770848, and NCT00422019).

Ficlatuzumab (AV-299) is a humanized monoclonal antibody against HGF developed by AVEO Pharmaceuticals (Cambridge, MA, USA) [192]. It is a neutralizing antibody that inhibits the biological activity of HGF and c-MET [192]. In the H596 NSCLC xenograft model, the anti-tumor efficacy of ficlatuzumab was verified and increased with combination therapy with gefitinib and cetuximab, an EGFR inhibitor, compared with single agent treatment [178]. Based on this evidence, clinical trials were initiated to investigate the efficacy of ficlatuzumab for head and neck squamous cell carcinoma, advanced solid tumors, and NSCLC [192,193,194]. Preliminary anti-tumor activity and manageable adverse events were observed in phase I clinical trials for advanced solid tumors, and a dose of 20 mg/kg every two weeks was recommended for phase II trials [193]. Currently, a randomized phase II study with gefitinib alone or in combination with ficlatuzumab is underway for the treatment of NSCLC, but significant benefits of the combination therapy have not yet been shown [192].

TAK-701 (L2G7) is a humanized monoclonal antibody developed by Galaxy Biotech (Sunnyvale, CA, USA) and derived from murine L2G7 monoclonal antibodies with high affinity for HGF [195]. Preclinical studies over the last few years have reported its anti-tumor efficacy. In particular, the combination treatment of TAK-701 and gefitinib markedly reduces tumor growth in HCC827-HGF cell xenografts, a human NSCLC cell line with an activating EGFR mutation and HGF overexpression; this treatment also inhibited phosphorylation of c-MET, EGFR, ERK, and Akt [195]. A phase I clinical trial has been completed to test the safety, tolerability, and pharmacokinetics of TAK-701 in patients with advanced solid tumors, resulting no specific dose-limiting toxicities [196].

Onartuzumab (MetMAB) is a humanized monovalent monoclonal antibody against c-MET developed by Genentech (San Francisco, CA, USA) [197]. It was generated using Knob-into-hole technology, which allows antibody binding to its receptor in a one-to-one fashion [197]. Onartuxumab inhibits the binding of HGF and suppresses the ligand-induced phosphorylation of c-MET. Preclinical studies have demonstrated that onartuxumab significantly inhibits tumor growth in xenograft models of glioblastoma without having a detrimental impact on body weight [197]. In a phase II study, despite clinical efficacy of onartuxumab plus erlotinib in patients with MET-positive NSCLC, it had no efficacy in a randomized phase III study [198,199]. Nevertheless, other clinical trials for patients with glioblastoma, gastric cancer, hepatocellular carcinoma, and breast cancer have been completed (Clinical trials information: NCT01632228, NCT01662869, NCT01897038, and NCT01186991).

Emibetuzumab (LY-2875358) is a humanized bivalent monoclonal anti-c-MET antibody developed by Eli Lilly (Indianapolis, IN, USA) [200]. Emibetuzumab has internalization activities that depletes c-MET from the cell surface, preventing HGF binding to c-MET [201]. Emibetuzumab has significant anti-tumor efficacy in in vivo HGF-dependent glioma and HGF-independent gastric and NSCLC xenograft tumors [201]. In phase I trials for patients with NSCLC, treatment with emibetuzumab alone or in combination with erlotinib had no dose-limiting toxicities or serious adverse effects at a dose of 2000 mg once every two weeks; a dose of 750 mg once every two weeks was recommended for phase II trials, which were completed in 2015 for gastric cancer [200].

## 6. CLEC14a Signaling

### 6.1. Overview

CLEC14a (C-type lectin domain family 14 member) is a member of the C-type lectin/C-type lectin-like domain (CTL/CTLD) superfamily, which also includes thrombomodulin, CD93, and endosialin [202]. It is a type I transmembrane protein composed of a signal peptide (amino acids 1–22), an extracellular domain (amino acids 23–398) containing a CTLD, an EGF-like domain, a low compositional complexity region, a transmembrane domain (amino acids 399–421), and a cytosolic domain (422–490) [203]. In 2011, it was first reported that CLEC14a is expressed exclusively in endothelial cells, and plays a key role in endothelial migration, tube formation, and endothelial cell-cell contacts in angiogenesis [204]. In addition, we also reported that the CLEC14a CTLD is important for regulating filopodia formation and cell migration [205]. 

To date, several CLEC14a-binding partners, including multimerin 2 (MMRN2; endoglyx-1) and heat shock protein 70-1A (HSP70-1A), have been identified. MMRN2 is an extracellular matrix glycoprotein that suppresses angiogenesis via direct binding to VEGF-A, and binds the extracellular domain of CLEC14a [206]. Inhibition of CLEC14a-MMRN2 binding suppresses sprouting angiogenesis and tumor growth [207]. HSP70-1A is a molecular chaperone that stabilizes newly synthesized or misfolded proteins inside cells. We found that extracellular HSP70-1A binds to the CLEC14a CTLD on the surface of endothelial cells and promotes CLEC14a-CTLD-mediated endothelial cell-cell contacts, suggesting that extracellular HSP70-1A secreted from tumor cells may stabilize CLEC14a to stimulate endothelial cell-cell contacts in angiogenesis [208].

### 6.2. Role and Relevance of CLEC14a in Cancer

In 2012, Mura et al. first identified CLEC14a as a novel tumor endothelial marker protein in patient tissue samples [203]. CLEC14a is exclusively expressed on tumor blood vessels in various cancers, including ovarian, liver, bladder, breast, kidney, pancreas, stomach, and esophageal cancer, but not on normal blood vessels. In the same paper, using the ectopic expression and siRNA-mediated knockdown of CLEC14a, the authors showed that CLEC14a regulates angiogenic functions including filopodia formation, endothelial cell migration, and tube formation, demonstrating the importance of CLEC14a in tumor angiogenesis. Furthermore, their findings showed that the expression of CLEC14a is regulated by shear stress, suggesting that low shear stress at the tumor endothelial surface may be one of factors leading to CLEC14a expression on tumor vessels.

Interestingly, CLEC14a is also highly expressed on blood vessels in RIP-Tag2 insulinoma and HPV16/E2-induced invasive cervical cancer [209]. In addition, CLEC14A mRNA is highly upregulated in CD109+ circulating endothelial cells in breast cancer and glioblastoma patients [210]. Recently, Noy et al. reported that tumor growth and vascularity was reduced in a CLEC14a-knockout syngeneic tumor mouse model, compared to wild-type mice [207]. More recently, Krishna et al., reported that microvessel density, detected using an anti-CLEC14a antibody, was significantly reduced in patients with epithelial ovarian cancer treated with chemotherapy, suggesting that CLEC14a may be a more specific endothelial marker to assess tumor angiogenesis [211]. Collectively, this evidence suggests that CLEC14a may be a novel tumor endothelial cell-specific marker of tumor angiogenesis and a central regulator of tumor angiogenesis.

### 6.3. Monoclonal Antibodies Targeting CLEC14a

Mura et al. first provided the proof-of-concept that antibody-based modulation of CLEC14a may be effective for suppressing angiogenesis. They showed that CLEC14a polyclonal antibodies inhibit angiogenic functions, such as cell migration and tube formation, in vitro [203]. The same group also developed a mouse monoclonal antibody (C4 clone) that binds to CLEC14a and inhibits tube formation and sprouting angiogenesis in vitro and in vivo [207]. They also showed that the C4 clone suppressed tumor angiogenesis and tumor growth by blocking the interaction between MMRN2 and CLEC14a. However, the identification of the C4 epitope on CLEC14a and its immunogenicity for therapeutic use require further study.

Since the CTLD is an important domain for CLEC14a-induced angiogenesis, we selected it as a target antigen to generate human monoclonal antibodies from a human synthetic single antibody library using phage display technology. We generated a human monoclonal antibody targeting the CLEC14a CTLD that has cross-species reactivity for human and mouse CLEC14a, and potently inhibits cell-cell adhesion, endothelial migration, and tube formation [205]. In the same study, we also demonstrated that it acts as an interaction blockade to inhibit CLEC14a-CTLD-mediated cell-cell contacts by blocking the interaction between CTLDs. Additionally, CLEC14a cross-linking with the antibody induces internalization and downregulation of CLEC14a expression on the surface of endothelial cells to suppress angiogenesis.

Our recent results demonstrate that an optimized human monoclonal antibody targeting the CLEC14a CTLD inhibits VEGF-dependent tube formation in vitro, rat aorta vessel sprouting, and microvessel formation in vivo. In addition, it does not affect VEGF signaling on normal endothelial cells, which are associated with bevacizumab side effects. Furthermore, this antibody suppresses tumor angiogenesis in the in vivo angiogenesis mouse models of SNU182 hepatocellular carcinoma, CFPAC-1 pancreatic cancer, and U87 glioma cell lines. Interestingly, we also found that our antibody not only effectively suppresses tumor angiogenesis in the in vivo angiogenesis mouse models of HCT116 colorectal cancer cell lines, but also in those of bevacizumab-resistant HCT116-derived tumor cells, suggesting that antibody targeting of the CLEC14a CTLD may be an effective strategy to improve treatment options for bevacizumab-resistant tumors [212]. Together, these data suggest that CLEC14a is a novel therapeutic target for tumor angiogenesis, and that antibody-based targeting of CLEC14a may be an effective strategy for suppressing tumor angiogenesis.

## 7. Conclusions

For years, anti-angiogenesis strategies using monoclonal antibodies have been effective in suppressing tumor progression and metastasis in combination with chemotherapy. In particular, bevacizumab was a best-selling product among oncology products worldwide in 2016. This review discusses the role and relevance of VEGF and VEGFR and other anti-angiogenic targets in cancer and the developmental status of monoclonal antibodies against these targets. Although this review mainly covers emerging anti-angiogenic targets in cancers, further studies to identify novel therapeutic targets are still important for the better clinical outcome of cancer patients. Furthermore, the recent advent of the bevacizumab patent expiration will support the development of a variety of bevacizumab-like anti-angiogenic antibodies, which include bispecific antibodies that target VEGF, as well as monospecific antibodies to be used in combination with bevacizumab. In addition, for the continuous improvement of patient survival and quality of life, the next-generation anti-angiogenic therapeutic antibodies must overcome the unmet medical needs of bevacizumab in terms of its adverse effects and drug resistance.

## Figures and Tables

**Table 1:** Selected examples of monoclonal antibodies currently in clinical use and development.

Target	Antibody	Company/Institute	Antibody Type	Status	Indication
VEGF-A	Bevacizumab (Avastin)	Genentech	Humanized, IgG1	FDA approval	Colorectal cancer, NSCLC, glioblastoma, breast cancer, renal carcinoma, epithelial ovarian cancer
VEGF-A, -B, PGF	Aflibercept (Zaltrap)	Regeneron	Fully human, IgG1	FDA approval	Colorectal cancer
VEGFR2	Ramucirumab (Cyramza)	Imclone	Fully human, IgG1	FDA approval, Phase II	Advanced gastric or gastro-esophageal junction adenocarcinoma, NSCLC, advanced or metastatic urothelial carcinoma
VEGFR2	Tanibirumab	Pharmabcine	Fully human, IgG1	Phase II	Recurrent glioblastoma
PDGFRα	Olaratumab (Lartruvo)	Eli Lilly	Fully human, IgG1	FDA approval	Soft tissue sarcoma
Ang-2	Nesvacumab (REGN910)	Regeneron	Fully human, IgG1	Phase I	Advanced solid tumors
Ang-1, -2	AMG780	Amgen	Fully human, IgG2	Phase I	Advanced solid tumors
Ang-2	MEDI3617	MedImmune LLC	Fully human, IgG1	Phase I	Advanced solid tumors
Ang-2 and VEGF	Vanucizumab	Genentech	Bispecific, IgG1	Phase II	Colorectal cancer
HGF	Rilotumumab (AMG102)	Amgen	Fully human, IgG2	Phase I, II, III	Gastric and esophagogastric junction cancer, NSCLC, metastatic colorectal cancer, prostate cancer, renal cell carcinoma
HGF	Ficlatuzumab (SCH900105)	AVEO Pharmaceuticals	Humanized, IgG1	Phase I, II	Head and neck squamous carcinoma, NSCLC
HGF	TAK-701	Galaxy Biotech	Humanized, IgG1	Phase I	Advanced solid tumors
Tyrosine-protein kinase Met (c-MET)	Onartuzumab (MetMab)	Genentech	Humanized, IgG1	Phase I, II, III	NSCLC, glioblastoma, gastric cancer, hepatocellular carcinoma, breast cancer
c-MET	Emibetuzumab (LY-2875358)	Eli Lilly	Humanized, IgG4	Phase II	NSCLC, gastric cancer
CLEC14a	-	Univ. of Birmingham	Mouse	Preclinical	Undetermined as yet
CLEC14a	-	Scripps Korea Antibody Institute	Fully human, IgG1	Preclinical	Undetermined as yet

## Abbreviations

Ang	Angiopoietin
CLEC14a	C-type domain family 14 member
CTLD	C-type lectin-like domain
HGF	Hepatocyte growth factor
Ig	Immunoglobulin
PDGF	Platelet-derived growth factor
Tie	Tyrosine kinase with Ig-like and EGF-like
VEGF	Vascular endothelial growth factor
